# Recommendation of Workplaces in a Coworking Building: A Cyber-Physical Approach Supported by a Context-Aware Multi-Agent System

**DOI:** 10.3390/s20123597

**Published:** 2020-06-25

**Authors:** Luis Gomes, Carlos Almeida, Zita Vale

**Affiliations:** 1 GECAD-Research Group on Intelligent Engineering and Computing for Advanced Innovation and Development, Polytechnic of Porto (P.PORTO), P-4200-072 Porto, Portugal; 1161304@isep.ipp.pt; 2 Polytechnic of Porto (P.PORTO), P-4200-072 Porto, Portugal; zav@isep.ipp.pt

**Keywords:** context-aware recommender systems, pre-filtering, fuzzy logic, multi-agent system, multi-armed bandit

## Abstract

Recommender systems are able to suggest the most suitable items to a given user, taking into account the user’s and item`s data. Currently, these systems are offered almost everywhere in the online world, such as in e-commerce websites, newsletters, or video platforms. To improve recommendations, the user’s context should be considered to provide more accurate algorithms able to achieve higher payoffs. In this paper, we propose a pre-filtering recommendation system that considers the context of a coworking building and suggests the best workplaces to a user. A cyber-physical context-aware multi-agent system is used to monitor the building and feed the pre-filtering process using fuzzy logic. Recommendations are made by a multi-armed bandit algorithm, using ϵ-greedy and upper confidence bound methods. The paper presents the main results of simulations for one, two, three, and five years to illustrate the use of the proposed system.

## 1. Introduction

The recommendation of items to users in commerce is not something new and already existed long before the invention of computers. From the seller perspective, good recommendation means higher profits and improving users’ engagement. Recommender systems have gained prominence in e-commerce platforms as an efficient solution to increase sales and customer engagement [1]. In Lu et al. [2], content-based collaborative filtering is proposed to recommend news for users on the home page of the search engine Bing. Netflix is a good example of a company that relies on recommendations, giving precious help to users [3]. The algorithms that they use can engage the user and increase user experience. Another promising application area where contextual recommender systems are getting applied is tourism [4,5,6,7].

A context-aware system can acquire, interpret, and use context information to adapt its functionality to the current context of use to provide the right services to a specific event, time, place, people, etc. This type of system is concerned with the use of contextual information to supply better services to the user [8,9]. The consideration of the context in recommender systems is desirable and vital to produce good quality recommendations with higher payouts.

Recommender systems are able to match users to items, where an item is a general term used to describe what it will be recommended to a user [10]. Items can be almost anything—in the case of the work presented in the current paper, an item is a workplace inside a coworking building. The proposed system matches the user needs with the workplace’s characterization and real-time context.

This paper proposes a novel context-aware recommender system using a pre-filtering process with fuzzy data collected by a context-aware multi-agent system (MAS) that monitors a coworking building. The use of a cyber-physical MAS allows real-time monitoring of context data inside the coworking building, to be later fuzzified and used in the pre-filtering process. This combination of MAS, fuzzy data, and pre-filtering enables the development of a novel context-aware recommender system that can recommend workplaces inside the coworking building. The proposed system overcomes existing systems by combining real-time data, fuzzification, pre-filtering, a multi-armed bandit algorithm, and user feedback. A multi-armed bandit algorithm is used with ϵ-greedy, and upper confidence bound (UCB) methods. The proposed solution was tested taking into account the multi-armed bandit algorithm method and data for several periods: one, two, three, and five years. This paper contributes to the integration of cyber-physical systems in a context-aware recommender system to provide efficient recommendations to the user. Being a cyber-physical recommender system, the paper also proposes a reward mechanism using mandatory user feedback that will decide the reward.

This paper is organized as follows: after this first introductory section, Section 2 presents related work already published. The context-aware multi-agent system is presented in Section 3. Section 4 presents the cyber-physical recommendation system with the processes that are involved: fuzzy logic application, pre-filtering process, multi-armed bandit algorithm, and user’s feedback. The results of the proposed solution are presented in Section 5, and Section 6 presents the main conclusions.

## 2. Recommender Systems

A recommender system is able to give recommendations to a user by considering several aspects—the user’s profile, item description, and context. Users can use several techniques and each problem should be analyzed individually. The design and development of a recommender system are neither straightforward nor a one-time process. Algorithms should improve continuously and experimentation techniques with users must be implemented to allow for the improvement of the algorithm [3].

Generally, there are three types of recommender systems: collaborative filtering, content-based, and hybrid systems [10]. The collaborative filtering matches users with similar tastes, while the content-based uses the user’s profiles and item’s description in order to make recommendations [11]. Both approaches can be used together in a hybrid system, as can be seen in Lian et al. [12], where a content-aware collaborative filtering system is applied to recommend points of interest according to user past actions, points of interest categories, and user profiles, and in Yang et al. [13], where a job recommendation system is proposed using an efficient statistical–relational learning approach.

In a recommender system, the use of hierarchies allows for the representation of the user’s context and the item’s context. This representation enables an easy understanding and a match among hierarchies [4,6]. These hierarchies can be built manually or dynamically [14].

Initial recommender systems allowed a match between users and items, disregarding the context. However, the context should be taken into account to improve the recommendations made to the users [15]. The use of context in recommender systems can be done as pre-filtering, contextual modeling, or post-filtering [16]. The pre-filtering approach uses an initial context filtering where terms and inputs are filtered according to the context, and only after this filtration, the recommendation will be made. The contextual model approach uses the context information directly in the model. The post-filtering approach initially executes the recommender system and then applies the context filtering on the results. A contextual recommender system using the contextual model approach is proposed in Song et al. [17], where a multi-arm bandit approach is used and where the evaluation of the payoff considers the context. In many applications, the huge number of possible context scenarios is a challenge to the system performance, and it is not possible to ensure the execution of the recommendation system for all the contexts [17]. Algorithms capable of dealing with a large set of data can also be used in recommender systems to address this problem [7].

The use of contextual data increases the number of possible scenarios in a system and can also increase the imprecision and uncertainty [11]. If the recommender system uses temperature as contextual data, and if the user wants a food plate that it is served hot, this brings ambiguity to the system, which may not know the practical meaning of being hot, too hot, warm, or normal. This can be solved using fuzzy logic. In Thong and Son [18], fuzzy logic is applied to medical symptoms to provide medical diagnosis recommendations. Fuzzy logic can also be used to combine contextual hierarchies, as seen in Wu et al. [19], which focuses on business-to-business e-services.

In addition to using context for recommender systems, sentiments can also be used to extract terms and analyze the semantics of users’ publications in social media [4]. This can contribute to a better recommendation system, but it demands a more complex type of feedback, where the user can freely report on social media. Text can be analyzed and converted to topic hierarchies, as seen in Sundermann et al. [14], where the LUPI-based incremental hierarchical clustering algorithm is used to analyze the webpage’s text.

Multi-agent systems (MAS) can also be used to benefit recommender systems [20,21,22]. MAS are commonly used as contributors or enablers for the recommender systems or contextual data acquisition, and not to make direct recommendations to the user.

## 3. Context-Aware Multi-Agent System

A multi-agent system (MAS) is composed of multiple agents that behave autonomously, but at the same time interact with each other, in order to perform tasks and to achieve a set of goals; they use their intelligence to accomplish the goals and react to environment changes [23]. On these systems, each agent has limited resources or incomplete information, so it is restricted in its capabilities, promoting decentralized systems and asynchronous communications [24].

According to Wooldridge, “an agent is a computer system that is situated in some environment, and that is capable of autonomous action in order to meet its delegated objectives” [25]. Wooldridge also described the abilities that an agent should implement: reactivity, proactiveness, and social. These abilities allow each agent to be context-aware, autonomous, and communicative [25].

Like human organizations, a multi-agent system possesses a variety of roles, relationships, and authority structures that manage its behavior. Consequently, all systems have some form of organization, explicit or implicit, formal or informal [26]. For this, effective communication is necessary among agents, to ensure the correct exchange of information.

The proposed context-aware multi-agent system provides contextual data to the recommender system. For this purpose, there are different sets of agents assigned to different tasks, but with the same main goal, which is understanding the surrounding environment. To achieve this, the use of sensors is crucial. Sensors enable extensive monitoring and better knowledge regarding the environment where they are deployed. In this work, three sensors are used: a light-dependent resistor (LDR) for light sensing, a sound detection sensor (microphone with an LMV324), and a DHT22 sensor for temperature and humidity readings. In the proposed system, each agent is devoted to a single sensor, with a one-to-one agent–sensor relation. Therefore, the type of agent is related to the respective sensor hardware functionality and agents must agree to work together towards a common goal.

According to Horling and Lesser, various types of agent organizations can be defined [26]. However, in this work, the proposed MAS does not fit neatly into a particular category; therefore, it must include characteristics of several different organization types—federations for data flow, teams for understanding the environment, and congregations—in order to provide additional benefits to the system. In practical terms, in each room of the coworking building, a federated organization is settled, which means that a group of agents gives some autonomy to a single delegate which represents the group. Group members interact only with this agent; therefore, it acts as an intermediary between the room group and the outside world, such as other federations and the recommender system. Inside of each room/federation, these multiple cooperative agents have the mission to understand the local environment. However, it is possible to have more than one agent of the same type, which are organized together as a congregation. This makes the system have congregation-like characteristics, where agents are aggregated according to their type/sensor. For example, a room with three temperature sensors has a congregation of temperature-type agents.

**Figure 1:**
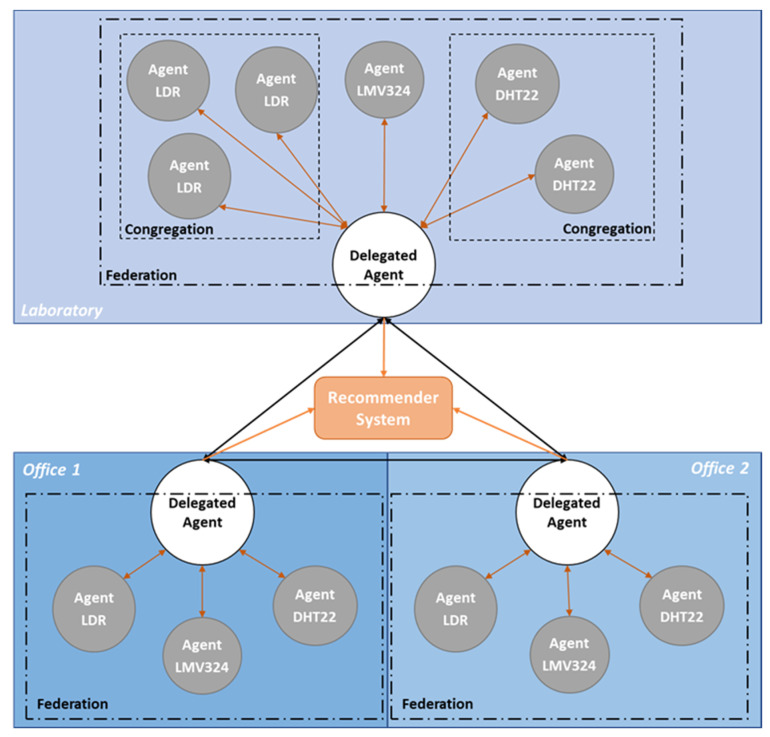
Multi-agent system organizations.

In other words, inside each federation there are multiple normal agents and one delegated agent. These delegated agents represent several rooms, which means that the coworking building is represented by several delegated agents. Figure 1 shows the organization of the proposed MAS.

One of the most interesting characteristics of this multi-agent system is its capability of extending the number of agents without additional manual agent configuration. These agents can learn from others, allowing the self-configuration of new agents according to the configuration of already deployed agents in the same federation. The proposed MAS is also scalable in the number of sensor types allowed. New sensors can be added to monitor new parameters, such as air quality and humidity. The agents use a mesh network that allows the connection of 255 nodes, enabling the installation of up to 254 sensors per office; one agent must be a delegated agent that does not have any sensor connected. However, this can compromise the performance of the delegated agent that must process and communicate 254 sensor readings, which can cause the filling of the communication buffer and consequently loss of data. In our experimentations, not more than 10 sensors were connected to a single delegated agent.

**Figure 2:**
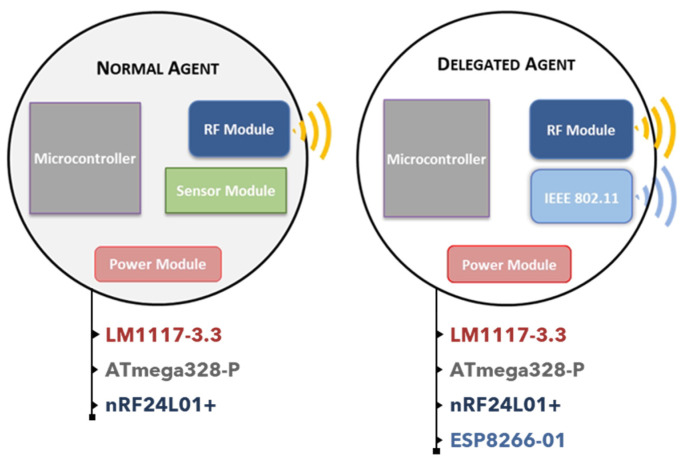
Hardware agents’ constitution.

In terms of hardware, there are two types of agents, as seen in Figure 2 normal agents and delegated agents. They both have a microcontroller, a power source, and a communication module (radio-frequency transceiver module) to ensure communication among agents. The normal agents have another component, the sensor module, essential to monitor environmental data. The delegated agents have an IEEE 802.11 module to communicate with the recommender system using transmission control protocol and internet protocol (TCP/IP) messages through Wi-Fi. Normal agents are composed by an LM1117-3.3 to reduce the power supply of 5 V/DC to 3.3 V/DC, the same ATmega328-P used in Arduino Uno with Arduino bootloader; an nRF24L01+ module for radiofrequency communications in a mesh network; and a single sensor that can be analog or digital. The delegated agent is composed by an LM1117-3.3, an ATmega2560 with Arduino bootloader; an nRF24L01+; and an ESP8266-01 for wireless IEEE 802.11 communications using TCP/IP. The microcontrollers use the serial peripheral interface (SPI) to communicate with the nRF24L01+. The delegated agent uses serial communication to communicate with the ESP8266-01 and has a more powerful microcontroller to enable handling multiple communications with normal agents.

The proposed context-aware MAS allows for the real-time monitoring of the building’s workplaces. The monitored data are needed for the recommendations system to match the user with the most appropriate workplace according to his or her needs and preferences. However, this MAS can be replaced by other monitoring systems, such as a supervisory control and data acquisition (SCADA) system.

## 4. Cyber-Physical Recommendation System

The problem addressed is the distribution of users/members in a coworking building with 15 workplaces: 3 meeting places, 2 laboratories, and 11 offices. All members have access cards that they use at the entrance of the building. The system must recognize the member and redirect him or her to the most appropriate workplace. When passing his or her access card, the member must specify the type of work: office work, meeting, or laboratory work. Then, according to his or her needs, profile, and workplace context, the system will recommend two workplaces to the user.

The proposed recommendation system is a content-based filtering that uses real-time contextual data from the MAS described in Section 3. In our solution, an item is a place (i.e., meeting room, laboratory, or office) that is recommended to the member to conduct his or her work. The payoff is given by the members’ feedback.

### 4.1. Fuzzification of Contextual Data

**Figure 3:**
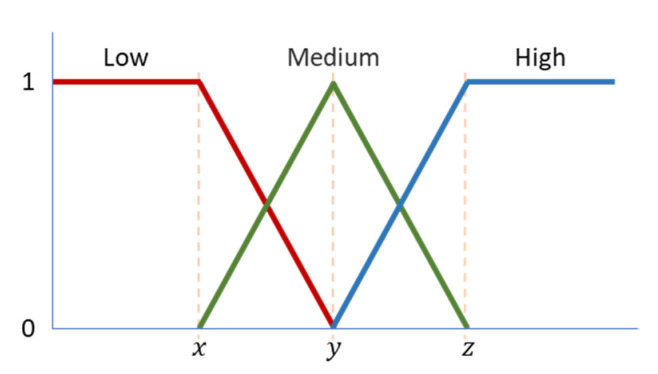
Fuzzy values representation.

The fifteen workplaces available in the coworking building have noise sensors, temperature sensors, and brightness sensors. The data obtained provide contextual information regarding the workplaces and create infinite possible contexts. Therefore, to minimize the number of possible contexts, fuzzy logic is used to classify the workplace’s noise, temperature, and brightness. The fuzzification is done according to values set by the user. Figure 3 shows the three fuzzy memberships that are considered in our system: low, medium, and high. These three memberships are used to characterize the workplace’s noise, temperature, and brightness. The variables x, y, and z are set by the user at the user registration (e.g., for temperature, the user can define x=18 ∧ y=22 ∧ z=25) and can be adjusted over time using the user’s feedback. The variables x, y, and z define the moment when the core ends and the boundary occurs. In our system, the low, medium, and high values are interrelated by their boundaries.

The fuzzification provides the degree of memberships of numeric input. Although it is possible to have multiple membership degrees, the sum of these degrees is always equal to 1, e.g., 0.7 low and 0.3 medium for a temperature of 16.5 °C of x=15 and y=20. The fuzzification is used to characterize the workplaces and to allow a pre-filtering process. For instance, in the previous example, where the workplace temperature is 0.7 low and 0.3 medium, the workplace will not appear for users with a high-temperature preference.

### 4.2. Pre-Filtering

**Figure 4:**
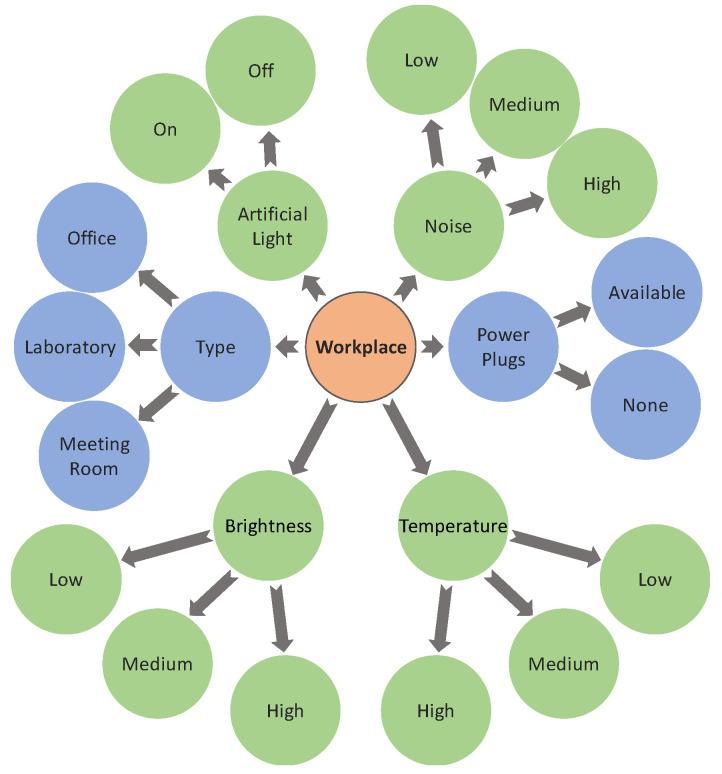
Workplace characterization.

In the proposed solution, a pre-filtering step is used to filter the available workplaces. The problem that our system tries to solve is whether a certain workplace inside the coworking building, under a particular context, should be recommended to a particular user. To solve this problem, the system needs to filter the available workplaces according to their context. In this filtering, two types of characterization are used: static and dynamic. Static parameters regard the constant characteristics of the workplace, e.g., workplace type, and the availability of power plugs. The dynamic parameters represent the current context of the workplace, which can change over time. Figure 4 shows the workplace characterization, in which green areas identify dynamic parameters and blue areas identify static parameters. The dynamic parameters were added taking into account the variables that can affect work productivity at the workplace: artificial light, level of noise, temperature, and light. These parameters were identified by Horr et al. [27], as physical environment factors that affect indoor environment quality impacting work productivity [27]. The parameter regarding air quality and ventilation, also identified by Horr et al. [27], was not considered in our proposed system because the authors were unable to find a proper office air quality sensor to be integrated into the MAS. Nonetheless, additional sensors can be added later to monitor other parameters.

The context-aware multi-agent system of Section 3 is responsible to get the dynamic parameters for each workplace. The “artificial light” parameter is a discrete variable while the rest of the dynamic parameters use the fuzzy approach to minimize the number of existing contexts. The contextual workplace description is a set of static and dynamic values:

(1) $$wd_{c}=\left\{ty,\;al,\;no,\;pp,\;te,\;br\right\},\;\;wd_{c}\in WP$$

where $wd_{c}$ represents the description of a workplace in context c, WP is the set of all workplaces, ty is the workplace type, al is the current state of artificial light, no is the noise average of last hour, pp indicates whether or not the workplace has available power plugs, te in the current temperature, and br is the current brightness of the workplace. To represent fuzzy membership degrees, variables no, te, and br can be represented as sets, e.g., br={(0.5, low), (0.5, medium)}.

The contextual workplace description is then matched with the user’s stored preferences and the user’s actual needs in real-time. The preferences define what the user likes, in terms of artificial light, noise, power plugs, temperature, and brightness. Users’ preferences are set by the user at the registration step, which allows them to overcome the initial recommendations of poor quality commonly associated with recommendation systems, known as a cold start [28].

The user’s needs refer to all the available parameters, i.e., workplace type, artificial light, noise, power plugs, temperature, and brightness. At the entrance of the coworking building, the user should choose the workplace type he or she needs. The user is also free to define any other parameter, updating their preferences. In the case that no definition is provided, the system will use the user’s predefined preferences. The final user’s needs are given by the combination of needs and preferences, as follows:

(2) $$u_{n}=\left\{ty,\;al,\;no,\;pp,\;te,\;br\right\}$$

The $u_{n}$ is matched to WP set to create the $WP_{m_{t}}$ set with all the workplaces that match the user needs at period t. Regarding fuzzy parameters, the match is validated by Equation (3). The $wd_{c}$ set can have a set of values representing noise, no; however, this is not true in the $u_{n}$, where no subsets are allowed:

(3) $$u_{n}\left\{need\right\}\;\wedge \;\left(x,\;level\right)\in wd_{c}\left\{need\right\}\;\forall \;x\geq 0.4$$

where need represents a fuzzy parameter and level the respective value. For instance, if the user needs a workplace with low noise (need=no ∧level=low), it is a match if the workplace has a no={(0.5, low), (0.5, medium)} and it is not a match if no={(0.3, low), (0.5, medium)}.

The proposed workplace characterization, seen in Figure 4, can be expanded to include new parameters monitored by new added sensors. This will create new possible contexts, but it will not negatively impact the fuzzification or the pre-filtering. The adding of new sensors in the proposed MAS will allow the recommender system to consider other parameters and provide better recommendations, but it could have an impact on the learning time.

### 4.3. Items Recommendation

The proposed recommender system is based on the multi-armed bandit problem where the system will try to maximize the cumulative reward $\sum_{t=1}^{T}r_{t}$, where $r_{t}$ is the reward of period t. In this paper, a period t is also defined as an iteration of the recommendation system, i.e., every time the user requires a recommendation. In the recommendation system, tuples are used to represent the workplace (W) and the workplace’s expected reward (R), creating tuples as (W, R).

The system uses a pre-filtering process that filters the available workplaces according to the context and the user preferences. The multi-armed bandit algorithm will try to maximize the reward using the $WP_{m_{t}}$ set and not WP. Therefore, the set of workplaces changes from iteration to iteration, making the top rewarded workplace not always achievable. The system uses a database that updates the rewards through time according to the available workplaces and user feedback.

**Figure 5:**
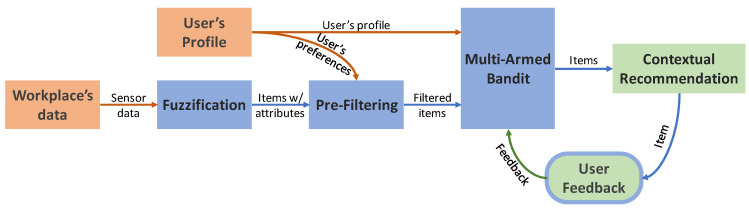
Recommender system overall.

Figure 5 shows the overall architecture of the proposed solution. The user profile is a set of static parameters set by the user. The workplace’s data are a combination of static and contextual parameters. The fuzzification process receives the workplace contextual data from the context-aware multi-agent system and converts sensor data to fuzzy membership degrees. The pre-filtering process combines workplaces with user needs and preferences. The filtered result and the users’ profile are inputs for the multi-armed bandit algorithm, and will result in two recommended workplaces, $W_{1_{t}}$, and $W_{2_{t}}$. The user can choose from these two possible recommended workplaces, being the final decision maker. As a reward, the system considers the user feedback and not the user’s decision (i.e., which workplace the user chose).

In our implementation, the multi-armed bandit uses two methods: ϵ-greedy, and upper confidence bound (UCB). The system does not run both methods at the same time; they were implemented for comparison purposes and to test the system. The ϵ-greedy method allows a random exploration of recommendations according to Equation (3), while the UCB provides a controllable exploration with low uncertainty, according to Equation (4):

(4) $$W_{1_{t}}=\{\begin{array}{c} max\left[Q_{t}\left(W\right)\right],\;if\;exploitation\;\\ E\left(W\right),\;\;\;\;\;\;\;\;\;\;\;\;if\;exploration \end{array},\;W\in \;WP_{m_{t}}$$

(5) $$W_{1_{t}}=max\left[Q_{t}\left(W\right)+c\sqrt{\frac{\ln\left(nr\right)}{N_{t}\left(W\right)}}\right],\;W\in WP_{m_{t}}$$

where t is the period, $Q_{t}\left(W\right)$ is the expected reward for workplace W at period t, c is the degree of exploration, nr is the number of recommendations already done by the system, and $N_{t}\left(W\right)$ is the number of times the workplace W was recommended prior to period t.

The proposed system provides two workplace recommendations. Therefore, a $W_{2_{t}}$ must be calculated. In ϵ-greedy method, the second-best is used for this purpose, in case of exploitation, and a random workplace, in case of exploration, complied with the restriction $W_{1_{t}}\neq W_{2_{t}}$. However, future developments could consider a higher ϵ to calculate $W_{2_{t}}$, or ϵ=0 to calculate $W_{1_{t}}$, prioritizing a higher exploration rate for the second recommendation, $W_{2_{t}}$. In the case of the UCB method, $W_{2_{t}}$ will have the second-best solution of Equation (4).

The multi-armed bandit algorithm enables the recommendation of items according to the previously achieved rewards, considering past user experiences. This paper proposes the multi-armed bandit, but other algorithms can be used, such as the k-nearest neighbors algorithm. The changing of the algorithm will not affect the proposed system where real-time data, fuzzification, pre-filtering, recommendation algorithm, and user feedback are used to create a contextual recommendation system to recommend workplaces to users in a coworking building.

### 4.4. User Feedback

The user feedback is used to improve the system’s performance. One of the main limitations of content-based filtering is the acquisition of feedback [9]. Therefore, the proposed system applies mandatory feedback every time the user wants to reuse the system. If the system detects that the user did not provide feedback regarding the last use, then the user must give feedback before using the system again. However, to not demotivate/disengage the user, this feedback step can be manually overpassed. In the proposed solution, the user’s feedback is used as the reward in the multi-armed bandit algorithm. This means that the reward may only be obtained days after when the user returns to the building and provides feedback. The proposed system attributes the reward to the workplace.

The user feedback ($fd_{ur}$) uses a four-level scale ($fd_{ur}\in \left\{-2,\;-1,\;1,\;2\right\}$), where −2 represents a bad experience, −1 unpleasant experience, 1 a pleasant experience, and 2 a good experience. For recommendations without feedback, meaning that they were manually overpassed, it is considered $fd_{ur}=0$. The four-level scale of the user’s feedback provides the reward value for the multi-armed bandit algorithm. 

## 5. Case Study and Results

This section uses a case study with a coworking building with 15 workplaces to illustrate the use of the proposed recommendation system. The case study is based on the building presented in Santos et al. [29], where a knowledge-based system is presented for intelligent control. In this paper, the proposed recommender system is used with both ϵ-greedy and UCB methods.

**Figure 6:**
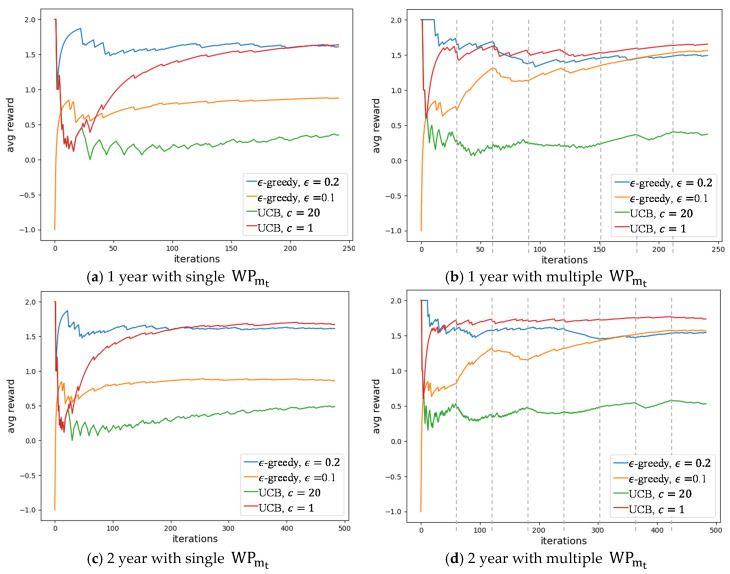

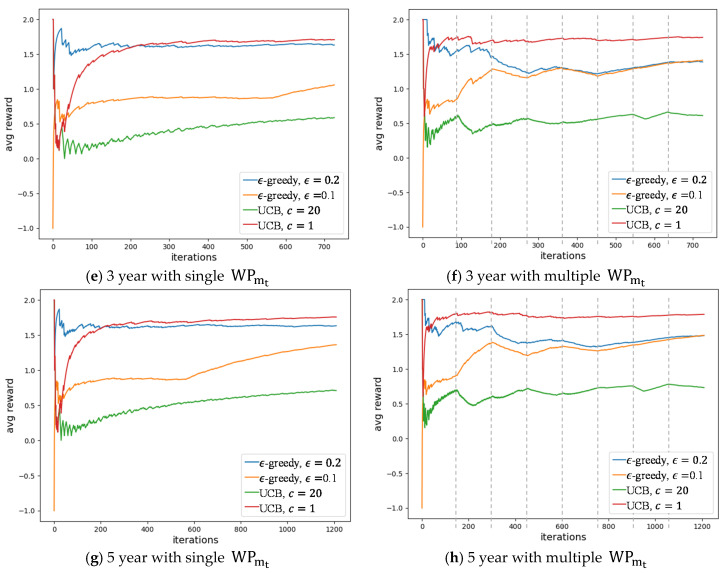
Results for the proposed recommender system using different time windows and contexts: (**a**) one year data using a single context; (**b**) one year data using eight contexts; (**c**) two years data using a single context; (**d**) two years data using eight contexts; (**e**) three years data using a single context; (**f**) three years data using eight contexts; (**g**) five years data using a single context; (**h**) five years data using eight contexts.

The proposed system was developed in Python and executed for four different time periods: one, two, three, and five years. It is considered that in one year the system is used 242 times, one time for each of the 22 working days in a month, multiplied by eleven working months. To check the contextual performance, each algorithm was executed for a scenario with a single context (i.e., constant $WP_{m_{t}}$, where $WP_{m_{t}}=WP$), and for a scenario with eight different contexts (i.e., eight $WP_{m_{t}}$, where $WP_{m_{t}}\in WP$). The contexts and user feedback were included in our simulation to validate if the average reward could achieve a value of 2, indicating a good experience. The simulations were made considering 15 workplaces where 3 represented good experiences to the user. Four algorithms were tested: ϵ-greedy with ϵ=10%, ϵ-greedy with ϵ=20%, UCB with c=1, and UCB with c=20. Figure 6 shows the results.

As seen before, the proposed algorithm recommends two workplaces inside the coworking building. For the test results presented in this section, it is considered that the user randomly chose one of the two recommended workplaces. The user feedback was done using static values representing the rewards per workplace, ranging for −2 to 2; the results consider that all the recommendations receive the user’s feedback.

For a single context, the UCB method with c=1 achieved the best results. According to the users’ feedback, the higher reward available is 2, meaning that the user had a good experience in the recommended workplace. The UCB method with c=1 achieved an average reward of 1.757 at the end of the fifth year. At the end of the first year, the same method achieved an average reward of 1.636. The 1.5 average was reached after 140 days. The ϵ-greedy with ϵ=20% presents a similar result at the end of the first year, 1.607, and it only took six days to have a result above 1.5. No other method was able to achieve values above 1.5. The UCB method with c=20 was, as expected, the worst method because of its high level of exploration. Simulations for 2 and 3 years presented similar results without significant improvement. However, in the third year, the ϵ-greedy with ϵ=10% was able to significantly improve the rewards. This could indicate overfitting during the first two years that was overcome with the exploration.

The eight contexts were equally distributed in a sequential manner in the time window; they are identified by dashed gray lines in Figure 6b,d,f,h. The UCB method with c=1 was also the best method for multiple contexts, an average reward of 1.653 in one year and 1.788 in five years. However, using a single year the results from the different methods were very close with the following average rewards: 1.653 for the UCB method with c=1, 0.376 for the UCB method with c=20, 1.562 for the ϵ-greedy method with ϵ=10%, and 1.492 for the ϵ-greedy method with ϵ=20%. It was in the three-year execution that the UCB method with c=1 showed a significant improvement. At the end of five years, the UCB method with c=1 was able to achieve an average reward of 1.788. This means that at the end of five years, the UCB method with c=1 had a better reward average for multiple contexts. For multiple contexts, the ϵ-greedy method also showed quality results, achieving average rewards of 1.481 and 1.483, for ϵ=20% and ϵ=10%, respectively. Despite presenting an average lower than the UCB method with c=1, the ϵ-greedy method with ϵ=10% was the only method with an uphill trajectory at the end of the fifth year.

In all simulations, it is possible to see the reward average achieving the maximum value possible, 2, indicating that the algorithm was able to recommend the best workplace possible at its first iteration. Over the next iterations, the algorithm will exploit new recommendations, and its average result decreases.

## 6. Conclusions

Recommender systems are a useful tool for user engagement. They should provide useful recommendations to the user and continuously learn from user interaction. For that, the context of the recommendation should be considered to improve the quality of the recommendations. This paper considers a coworking building and proposes a recommendation system to provide the user with suggestions regarding the best workplaces, considering the workplace context, user’s profile, and past user’s feedback.

The proposed methodology uses a combination of fuzzy logic, contextual pre-filtering, a multi-armed bandit algorithm, and a user’s feedback mechanism, all supported by a context-aware multi-agent system that monitors the building and the respective workplaces. The use and the results of the proposed solution are illustrated using different scenarios, contexts, and time windows. The results pointed out that, for this implementation, the upper confidence bound method with c=1 produces the best results.

The proposed solution recommends two workplaces for the user, and it is the user that makes the final choice. The results of the proposed solution are promising and confirm the feasibility and the interest of the practical use of such a solution. The integration of fuzzy logic allows a good pre-filtering of workplaces and reduces the context possibilities. Also, the application of the multi-armed bandit using the upper confidence bound method produces good quality results in an acceptable time window. Nevertheless, evolutions of the proposed solution are possible, namely using several methods and method configurations in the same system. For instance, the first recommendation could use upper confidence bound while the second could use the ϵ-greedy method. Another alternative is to use the same method but with different exploration and exploitation rates.
